# Challenges encountered by local health volunteers in early diagnosis and prompt treatment of malaria in Myanmar artemisinin resistance containment zones

**DOI:** 10.1186/s12936-016-1368-5

**Published:** 2016-06-06

**Authors:** Myat Htut Nyunt, Khin Myo Aye, Khin Thiri Kyaw, Soe Soe Han, Thin Thin Aye, Khin Thet Wai, Myat Phone Kyaw

**Affiliations:** Department of Medical Research, Yangon, Republic of the Union of Myanmar

**Keywords:** Malaria, Volunteer, Myanmar, MARC, Artemisinin resistance, RDT

## Abstract

**Background:**

After artemisinin resistance was reported, the Myanmar artemisinin resistance containment (MARC) project was initiated in 2011. One of the activities of MARC is to train volunteers for early diagnosis and prompt treatment by providing rapid diagnostic tests (RDT) and artemisinin combination therapy. This study aimed to fulfil the gap of information on the challenges faced by malaria volunteers in artemisinin-containment areas.

**Methods:**

A cross-sectional, descriptive study was conducted in 11 townships in MARC areas to assess the challenges in early diagnosis of malaria and treatment by malaria volunteers using qualitative and quantitative approaches.

**Results:**

Altogether 405 volunteers participated in the study. Although 97.5 % of volunteers can interpret a positive result for malaria, only 41.2 % correctly stated the persistence of a positive result in recently infected cases. Over 80 % knew the effects of temperature and humidity on performance of the malaria RDT. Unexpectedly, 15.1 % perceived that expired RDTs can still be useful for diagnosis although 98.3 % of respondents cited that the overall results of RDTs were reliable. Although most of them knew the treatment for malaria based on RDT results, some could not give the correct answer, while a few (2 %) mentioned artesunate monotherapy for RDT-negative cases. Training received by volunteers was also varied in study sites and 92.1 % believed that it was not sufficient. A certain portion of them faced the problem of regular supply of RDTs (9.9 %) and drugs (47.5 %), interpretation of result of RDTs (30 %), and performing blood test (20 %). The median RDT tested per month (25th, 75th percentile) was 6.0 (2.0, 15.0) indicating the need for prioritization based on endemicity. Regular reporting, supervision, monitoring system, and proper refresher training using uniform content of guideline to correct misconception of the volunteers, were needed to be strengthened. Moreover, the reliable and regular supply of materials and exchange system for expired RDTs and anti-malarials was important in the effectiveness of volunteers in MARC zones.

**Conclusions:**

Adequate refresher training, monitoring, supervision, and regular reliable supply of RDTs and anti-malarials were needed for capacity strengthening of volunteers in MARC zones.

## Background

Artemisinin-based combination therapy (ACT) is the first-line treatment for uncomplicated falciparum malaria in most endemic countries. Recent evidence of artemisinin-resistant *Plasmodium falciparum* in the Cambodia-Thailand border stimulated the launch of a multifaceted containment programme. This programme covered early diagnosis and appropriate treatment, decreasing drug pressure, optimizing vector control, targeting the mobile population, strengthening management and surveillance systems, and operational research [1].

Malaria-endemic countries should consider the following classification for implementation. This classification elucidates Tier I areas with credible evidence of artemisinin resistance, Tier II areas with significant inflows of people from Tier I areas, including those immediately bordering Tier I and III areas with no evidence of artemisinin resistance and limited contact with Tier I areas [2, 3].

In Myanmar, rapid diagnosis tests (RDTs) have been used for diagnosis of malaria in areas where there is no reliable microscopic facility, since the year 2000. *Plasmodium falciparum* is the main pathogen for malaria (75 %), followed by vivax infection (25 %); 152,195 cases have been reported in 2013 [4]. Combo RDT that can detect the PfHRP2 and pan-pLDH or vivax-specific pLDH devices have been distributed [5]. The implementation of RDTs commenced in 2003 in rural health centres and sub-centres in 284 endemic townships. About 400,000–500,000 tests have distributed annually, since 2006 [5]. A total of 887,969 and 1,587,745 fever cases were tested by RDT in 2012 and 2013, respectively. Local health volunteers are the major human resources for diagnosis of malaria using RDTs in containment areas [5]. However, challenges, including problems of distribution for widespread adoption of diagnostics by RDTs, have been reported in many other countries [6] as has variability in the quality and performance of RDTs, including sensitivity, specificity, heat stability, and longevity [7, 8]. Furthermore, evidence from other studies [9, 10] suggests that some providers and patients are unaware of the harm associated with treating cases without malaria with ACT, and in some settings, patients with negative results from a diagnostic test still receive and take anti-malarial medicine. This presumptive use of ACT may lead to unnecessary drug pressure and subsequently emergence and spread of drug resistance [11]. Moreover, over-usage of ACT causes unnecessary cost of anti-malarials and failure to treat other serious causes of fever [12].

In Tanzania, proper provider training and regular supervision encouraged compliance with testing results. In Ghana, in health facilities without previous access to malaria diagnostic testing, the introduction of RDTs significantly reduced the over-prescription of anti-malarials [13]. Nevertheless, ensuring the use of RDTs for the diagnosis of malaria and compliance with the results in the informal private sector is likely to be one of the challenges [3]. One study in Kenya [14] noted the demand for RDTs in the private sector at low prices, but access to RDTs only modestly improved targeting of ACT. The underlying reason was that most patients with negative test results purchased ACT anyway. Therefore, exploring the hidden problems and challenges in utilization of RDTs for diagnosis of malaria is essential for implementation of artemisinin containment measures.

In Myanmar, artemisinin resistance containment (MARC) areas trained local health volunteers to diagnose malaria by using RDTs and to provide ACT treatment [15]. Efforts are underway to improve the compliance of both providers and patients to the results of testing. The fifth objective of MARC is “to strengthen surveillance and operational research for development of evidence-based policies and strategies and for decision-making”. To fulfil this goal, there is a need to explore the challenges in diagnosis of malaria by RDT and treatment by using ACT among local health volunteers in MARC zones. There are potential links between the knowledge component of volunteers, including training sessions, refresher courses, attitudes, and perceptions and their practices related to RDTs, such as supply and support system, supervision, reporting and feed-back, and the effective utilization of RDTs and ACT by volunteers; this is the first study to fulfil the gap of knowledge after implementation of the volunteer programme in MARC zones.

## Methods

### Study design

A field-based, cross-sectional, analytical study with combined quantitative and qualitative approaches was conducted to assess the challenges encountered by malaria volunteers in MARC zones. The objectives of this study were to: identify the status of the utilization of RDTs in diagnosis of malaria by local health volunteers in MARC zones; to find out the knowledge, attitude and practices of health workers related to use of RDTs; to find out the barriers and constraints in diagnosis of malaria using RDTs and prescription of anti-malarials by malaria volunteers in field conditions.

### Study area and study population

**Fig. 1:**
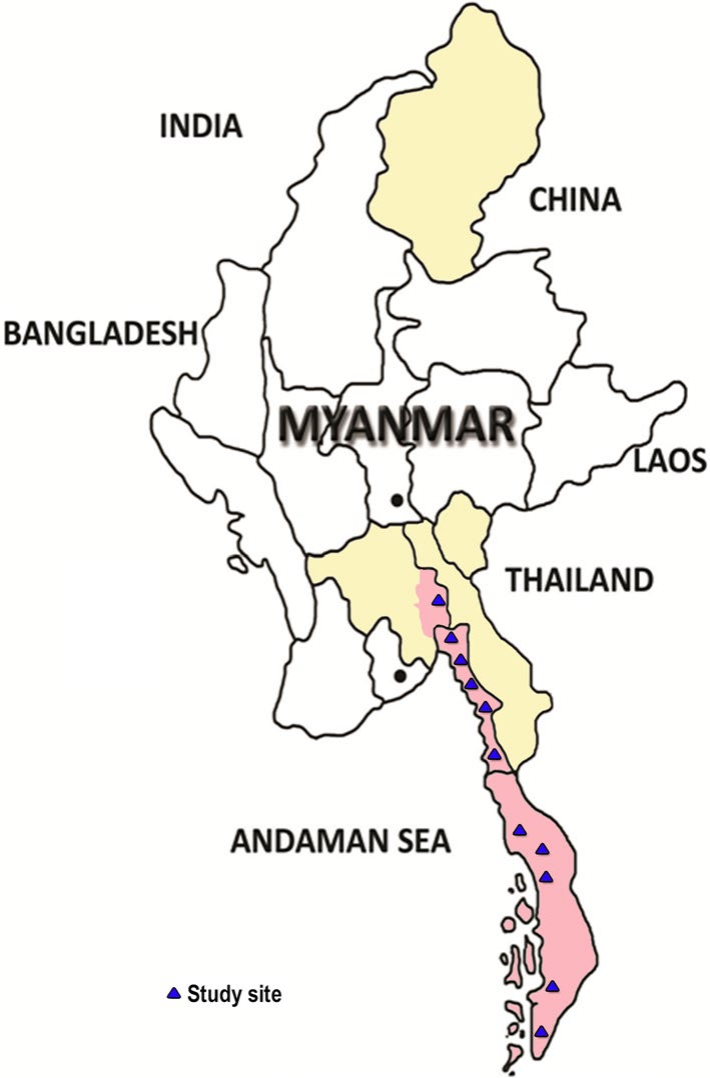
Study sites. *Light red* areas represent MARC zone I. *Light yellow* areas represent MARC zone II. Study townships are shown as *blue triangles*

This study was carried out in 11 townships, (five selected townships each from Tier I areas of MARC zones) that includes Tanintharyi Region, Mon State and Bago East Region (Fig. 1). The vector-borne disease control (VBDC) teams trained local health volunteers in these areas for early diagnosis of malaria by using RDTs and treatment by ACT. The number of local health volunteers varied in different regions depending upon the population and logistic parameters.

### Sampling procedure and sample size

In Myanmar, State or Regional Health Departments are composed of Township Health Departments that have rural health centres [5]. To get the representativeness for involvement of volunteers, a multistage, stratified, cluster-sampling technique was used. Five out of all townships in each State or Region of MARC Tier I were randomly selected for the study. If the number of townships was fewer than five, all the townships were included. The health centres of a selected township were listed and five health centres were randomly selected. If there were no more than five, all of the health centres were selected for the study. All local health volunteers under the jurisdiction of each selected health centre were recruited.

### Data collection methods

Trained interviewers used the pre-tested and modified structured interview questionnaires (SIQ) for face-to-face interviews of trained local health volunteers. The SIQ was designed to collect information on knowledge, attitude, perceptions, practices, barriers, and challenges on early diagnosis and treatment of malaria in MARC areas. For focus group discussion (FGD), six to eight trained local health volunteers were invited from three study sites in each township. Discussion guidelines comprised open-ended questions focusing on experiences of malaria diagnosis by RDTs, knowledge on treatment of malaria by ACT, feasibility in the study areas especially highlighting the importance of RDT use, and favourable and unfavourable conditions during implementation. The face-to-face interviews were conducted in volunteers’ homes in field sites using local language, and training materials, records of results and storage of RDTs and antimalarials were also requested.

Key informant interviews (KIIs) were carried out by using pre-tested guidelines for post-intervention assessment in MARC zones. Key informants included Regional Officers of VBDC teams of the study sites, all township Medical Officers from selected townships to explore the challenges encountered by malaria volunteers concerning their training, reporting, supervision, and monitoring.

### Data processing and analysis

Data collected during the survey were checked and entered into computer using Microsoft EXCEL and Epi Info version 7. The analysis was performed by SPSS version 20. Qualitative data were transcribed and analysed across themes and sub-themes for triangulation with quantitative data for meaningful interpretations.

### Ethical considerations

All respondents were clearly informed on the nature and the purpose of the study, private questions and benefits, right to refuse to participate or to withdraw from the study, and confidential handling of the data. Participation in this project was entirely voluntary. This project obtained ethical clearance from the Ethical Review Committee of the Department of Medical Research, Myanmar (ERC2/2011-24).

## Results

### Background characteristics

**Fig. 2:**
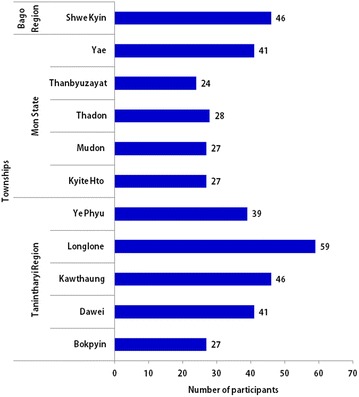
Number of participants from 11 townships in Tier I areas of MARC zone

In this study, a total of 405 volunteers were invited and all actively participated after giving informed consent. Among them, 42.9 % (172/405) of volunteers were trained by VBDC and 57.1 % (231/174) by non-governmental organizations (NGOs) that included Population Services International (PSI): 88 volunteers, World Vision Myanmar (WVM): 42 volunteers, International Organization for Migration (IOM): 42 volunteers, and Myanmar Health Assistant Association (MHAA): 46 volunteers. Around 3.2 % (13/405) of volunteers mentioned that they were trained by more than one organization. The MHAA alone was responsible for malaria volunteers in Shwegyin Township, Bago Region. The number of participants from different townships is shown in Fig. 2. Most of them (350/405, 86.5 %) were from the private sector (farmers, fishermen, manual workers, etc.). Only 13.5 % (55/405) of volunteers were government staff, such as teachers, officers, etc. Around 70 % (282/405) attained middle and high school level and only 10.4 % (42/405) primary school level.

### Knowledge on malaria RDT

Almost all respondents (99.5 %) knew that RDT is a blood test for malaria. Interestingly, (173/405, 42.7 %) could not mention the brand name of RDT that can detect the HRP2 alone or combination with pan-pLDH or vivax-specific pLDH, such as PARACHECK (191/405, 47.2 %), SD (130/405, 32.1 %), CARE START (120/405, 29.6 %), OPTIMAL (23/405, 5.7 %), and VESITEST (14/405, 3.5 %). Although most (395/405, 97.5 %) can interpret the positive result of RDT to indicate malaria infection and 315/405, 77.7 % noticed that partially treated cases may be positive result of RDT, only 167/405, 41.2 % knew that persistence of HRP2 can cause a positive result of RDT after treatment.

**Fig. 3:**
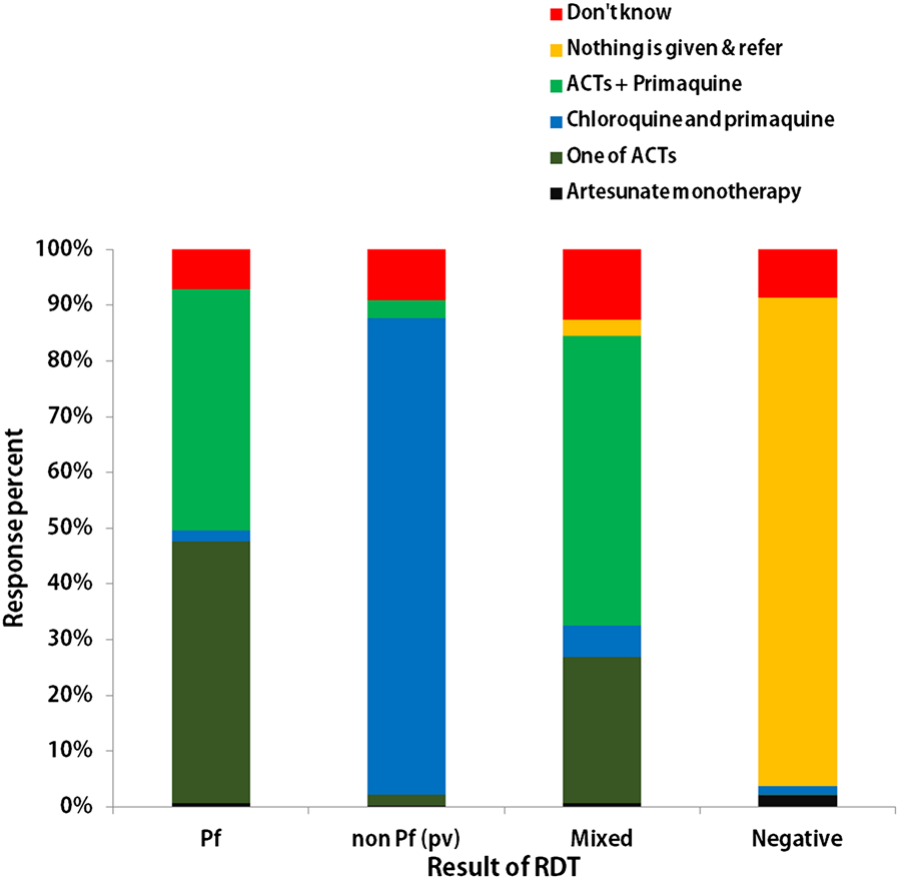
Knowledge on treatment of malaria based on RDT result by malaria volunteers

Regarding treatment of falciparum malaria, 46.9 % (190/405) answered ‘one of ACT for treatment’, another 43.2 % (175/405) mentioned ‘ACT followed by primaquine’, and only 7.2 % (29/405) did not remember the treatment. On treatment for vivax, mostly (85.4 %, 346/405) answered correctly the recommended treatment: chloroquine followed by primaquine. Unexpectedly, a few (8/405, 2 %) answered that artesunate monotherapy could be given for RDT-negative cases. Their responses for treatment of malaria based on the result of RDT are shown in Fig. 3.

As might be expected, training was the main source of information on the knowledge of RDT (388/405, 95.8 %), health staff (122/405, 30.1 %), and manuals and instructions (81/405, 20 %).

More than 80 % of respondents knew that temperature and humidity could affect the performance of RDTs. However, 15.1 % (61/405) mentioned that expired RDT can be useful for diagnosis. Most of them could interpret the positive result for diagnosis of malaria (395/405, 97.5 %). However, some (167/405, 41.23 %) mentioned that recently treated cases could provoke positive results. In addition, more than half of the respondents cited that past infection of malaria could be detected by RDT (Table 1).

**Table 1:** Knowledge and attitude of trained volunteers on interpretation of malaria RDTs

Result	Interpretation^a^	Frequency	Percent
Positive result	Malaria infection	395	97.53
	Recently treated cases	167	41.23
	Partially treated cases	315	77.78
Negative result	No malaria	388	95.80
	Past infection^b^	167	41.23
	Improper testing	224	55.31
	Expired or poor storage of RDT	273	67.41
Invalid result	Device error	353	87.16
	Operational error	185	45.68
Source of information	Training	388	95.80
	Health staff	122	30.12
	Books and instructions (manual)	81	20.00
	Medical sale personnel	3	0.74
RDT result is reliable	Yes	398	98.27
	No	7	1.73
If the RDT result is doubtful	Refer to health centres	191	47.16
	Try with another one more	195	48.15
	Try with another brand test	19	4.69

^a^Column percentages do not add up to 100 due to single item responses

^b^Past infection means history of malaria infection in patients that is not recently (>3 months)

### Attitudes towards the result of malaria RDT

Around 98 % felt that the results of malaria RDT were reliable. If the result was doubtful by volunteers (faint band, no control band, suspected fever cases with negative RDT result, etc.), 48 % would like to test another brand of RDT. Conversely, 47 % would like to refer immediately. Nearly 90 % mentioned that blood test for malaria could be done only after training. If they wanted to know more information, 95.5 % referred to their seniors and 60 % wanted to search the literature. Nearly 100 % understood that they should adhere to guidelines for blood testing and prescription of anti-malarials. Moreover, 92 % would like to refer immediately if a patient’s conditions worsened. Very few (6 %) wanted to wait and only 1.7 % wanted to change the drugs by themselves.

### Practices related to RDT

One-third of participating volunteers performed one to five blood tests per month. Nearly 15 % received no patients for blood testing. The median RDT tested per month (25th, 75th percentile) was 6.0 (2.0, 15.0) and positive RDT per month was 1 (0.0, 4.0) volunteers in previous month. Among them 16/405 (14.8 %) had conducted no blood tests in the past month while three volunteers tested up to 100 RDTs; exceptionally, one volunteer performed up to 200 RDTs. Unexpectedly, around 19 % experienced invalid results (no control band) and 23 % had encountered suspect results of RDT (faint band, unclear band, and fever with chills and rigours with negative RDT result). The number of volunteers who received training varied widely. Approximately 70.1 % of them received training at least one to two times. Around 92 % felt that the training provided was not sufficient for them. Nearly 10 % faced problems on regular supply of RDTs and drugs (47.5 %), interpretation of results of RDTs (30 %), and performing the blood test (20 %).

### Reporting and supervision

**Fig. 4:**
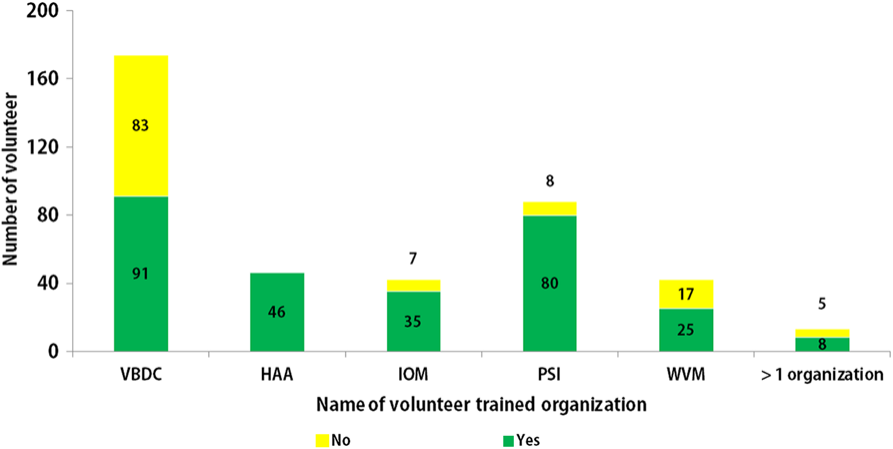
The status of supervision and monitoring of volunteers trained by different organizations

Around 91 % (369/405) of respondents sent their monthly reports regularly to the responsible organization. Of them, those who were trained by VBDC teams attained the highest rate (158/172, 91.9 %). There were variations in supervision and monitoring of volunteers by different organizations. The VBDC teams supervised and monitored 53 % (91/174) of volunteers but WVM monitored nearly 60 % (25/42) of volunteers. The IOM and PSI attained much higher rates (35/42, 83 % vs 80/88, 91 %). The rate of monitoring and supervision of volunteers by HAA was 100 %. Among the volunteers trained by more than one organization, nearly 62 % (8/13) received supervision and monitoring (Fig. 4).

### Qualitative findings

Excerpts from KIIs revealed the perceptions, opinions and challenges in volunteer training programmes and their use of RDTs and ACT. One of the VBDC staff expressed his opinion on training of volunteers:

Volunteer projects should be prioritized. More effort is necessary in endemic and really hard-to-reach areas than easily accessible areas.

One township Medical Officer (TMO) expressed his views on the training programme:

The training content and duration are not uniform for volunteers. It should be standardized across each and every organization involved. The training contents should be prioritized as nice to know, must know etc. and the level of understanding of trainers is also important.

Apart from the training, one volunteer highlighted the inadequate supply of materials compared to demand:

When I requested malaria RDT, about 20 tests per month were provided. It was not sufficient enough when I needed to handle more than 30 patients. In that case, I borrowed RDTs from others.

One volunteer shared his experience that he had not come across RDT-positive patients:

I wanted to see the positive result but not yet encountered. So, I had to avoid the provision of drugs to patients and just referred them to midwives.

One volunteer shared his usual practice:

When performing malaria RDTs, if the control line appears, it is still in good condition even after the expiry date.

Volunteers shared their experiences on challenges encountered in supplies of RDT and ACT and their ideas on improvement to the training programme:

Training should include short field trip to the village to practice with real patients.

One TMO complained about the reporting:

We request to send the report regularly but some volunteers do not send monthly.

One volunteer explained failure to send the monthly report regularly:

It is not easy to report monthly because sometimes, we did no blood test per month, difficulties in transportation, high cost for transportation to send the report, and no time to send the report.

Concerning supervision and monitoring, one VDBC staff shared his experience:

We know to perform the supervision and monitoring. But there is no specific budget allocation. So, we have to do together with other health activities.

## Discussion

There are reports of effectiveness of volunteers in control of communicable diseases [16, 17]. Conversely, one study mentioned that they did not adhere to their treatment plans in line with results of RDTs [9]. Another study from Sudan found that community volunteers prescribed ACT in 30 % of fever cases with negative RDT results [16]. The misdiagnosis of malaria can lead to severe disease and death, or unnecessary exposure to anti-malarial drugs, which may lead to drug toxicity and resistance and, in turn, lack of confidence in health care services [18].

In this study, although most can interpret an RDT result correctly, only 41 % of respondents were aware that ‘recently treated cases’ is one of the possible reasons for positive result, while about 55 % noticed that ‘improper testing’ may cause a negative result. Although pan-pLDH antigen band disappeared after treatment, persistence of the HRP2 antigen in recently treated patients was common [19] and may lead to misinterpretation of an RDT result. This means that the volunteer’s knowledge on the interpretation of the RDT result needed to be improved, especially on recurrent fever cases who had been diagnosed and treated for malaria recently.

In the present study, the overall knowledge of RDT by malaria volunteers was satisfactory, but misconceptions existed. Around 15 % of the respondents mentioned that expired RDTs could be useful for diagnosis of malaria and were not aware of the effect of temperature and humidity on malaria RDTs. Similar findings have been reported in one study [20] that observed only 9 % of participants checked the expiry date of RDTs and 24 % were unable to read the test correctly. In the present study, almost all respondents mentioned that the results of RDTs were reliable. If the result of a RDT was doubtful, 48 % wanted to test another one and another 47 % wanted to refer to hospital or other laboratory. A small portion (6 %) mentioned that they wanted to wait and see if the patient’s condition worsened. Although the general attitude of malaria volunteers towards the use of RDT and ACT was satisfactory, doubtful RDT results highlight the need for guidelines for suspect RDT results.

Nearly half of participants knew Coartem (ACT) for treatment of *P. falciparum* mono-infection. Only 43.2 % correctly mentioned a single dose of primaquine should be followed after ACT for *P. falciparum* mono-infection. Although only 2 % mentioned artesunate monotherapy for RDT-negative cases, it reflected the self-treatment of artesunate monotherapy in endemic areas. Their misconceptions required attention for arrangements of further refresher training.

This study found that 19 % of volunteers experienced invalid RDT results and 23 % had encountered suspected results of RDT, such as faint band, unclear band, and fever with chills and rigours with negative RDT results. This finding indicates the need for guidelines and instructions to volunteers on how to proceed with invalid or doubtful RDT results in the management of cases. Moreover, 30 % had the problem of interpretation of a result, such as invalid or confused on interpretation of the result of RDT. Repetitively negative results of an RDT was also reported in the qualitative findings and led to doubt in the quality and interpretation of the blood test, causing attrition in the volunteer.

The workload for performing the malaria RDT widely varied among volunteers. The median RDT tested per month (25th, 75th percentile) was 6.0 (2.0, 15.0). While few were performing more than 50–100 tests per month, some had no patients for blood testing or all negative results. This may be due to equal assignment of the volunteers regardless of the prevalence of the disease in different endemicity and local transmission. Priority assessment of the villages should be done based on accessibility to a health care centre, endemicity, malaria micro-stratification, and transportation. Villages close to health facilities should be omitted for volunteer training and unnecessary training of volunteers in irrelevant villages should be brought to a halt to avoid wastage of human resources, RDT and drugs. Some showed lack of knowledge for RDT-negative cases. Early referral systems should be encouraged in RDT-negative cases.

In this study, some experienced difficulties requesting materials. The reliable and regular supply of RDT and anti-malarials was important in implementation of the volunteer project. Although most of the volunteers sent their monthly reports regularly, many factors influencing regular reporting have been explored, such as no blood test per month, difficulties in transportation, high cost for transportation to send the report, and no adequate time to send the report. Supervision was still weak, especially for VBDC-trained volunteers. However, Myanmar HAA and PSI had highest supervision rates for their volunteers. Their staff collected the monthly reports and performed the supervision and monitoring in the villages where volunteers resided. Conversely, there was no specific budget allocation for supervision and monitoring, precluding long-term, regular supervision and monitoring of volunteers. The supervision component was to be strengthened for quality control and sustainability of the programme as also stated by one cluster, randomized, controlled study on volunteers in Bago Region, Myanmar [17].

## Conclusions

In Tier I areas of MARC zones, local health volunteers can carry out early diagnosis and prompt treatment of malaria effectively in remote sites. However, there were wide variations in the workload of volunteers concerned with confirmation of malaria by RDT. Prioritization of supplies of RDTs is required to be consistent with the prevalence of malaria in the locality. Furthermore, the reliable and regular supply of RDTs and anti-malarials is important for malaria control in remote sites. There are gaps in knowledge and misconception of volunteers that might hamper their performance in using RDTs for diagnosis of malaria and adequate treatment with ACT.

### Policy implications and recommendations

- The knowledge of the malaria volunteers on early diagnosis and prompt treatment of malaria needs improvement by proper refresher courses that should be uniform for all organizations involved in volunteer training and should correct common misconceptions of volunteers.
- Some volunteers needed more RDTs and anti-malarials, while some received more than enough. Priority assessment is essential for equity in supply of malaria RDTs and ACT.
- Regular and reliable supply of malaria RDTs and ACT, including the exchange system for expired tests.
- Weaknesses in supervision by the responsible authority, especially for volunteers trained by VBDC, can be alleviated through proper refresher training, monitoring and supervision in order to enhance performance.

## Abbreviations

ACT: artemisinin combination therapy
HAA: Health Assistant Association
IOM: International Organization for Migration
MARC: Myanmar artemisinin resistance containment
NGO: non-governmental organization
PSI: Population Service International
RDT: rapid diagnosis test
VBDC: vector-borne diseases control
WVM: World Vision Myanmar
